# Assessment of myocardial scarring improves risk stratification in patients evaluated for cardiac defibrillator implantation

**DOI:** 10.1186/1532-429X-13-S1-O100

**Published:** 2011-02-02

**Authors:** Igor Klem, Jonathan W Weinsaft, Bahnson Tristram, Don Hegland, Han W Kim, Brenda Hayes, Michele A Parker, Robert M Judd, Raymond J Kim

**Affiliations:** 1 Duke University Medical Center, Durham, NC, USA; 2 Weill Cornell Medical College, New York, NY, USA

## Objective

We hypothesized that an assessment of myocardial scarring by cardiac magnetic resonance (CMR) would improve risk stratification.

## Background

Current sudden cardiac death (SCD) risk stratification emphasizes left-ventricular ejection fraction (LVEF), however the majority of patients suffering SCD have a preserved LVEF and many with poor LVEF do not benefit from ICD prophylaxis.

## Methods

One hundred thirty-seven patients undergoing evaluation for possible ICD placement were prospectively enrolled and underwent CMR assessment of LVEF and scar. A comprehensive medical history including CAD risk factors, heart failure functional class (NYHA), and medications at the time of CMR was obtained in all patients. A total of 105 (77%) patients underwent EPS within a median of 0 days (IQR 0, 3.5) of CMR. No patient experienced a change in clinical status in the time between CMR and EPS. 103 patients (75%) had an ICD placed, generally during the initial evaluation, 2 days (IQR 1, 7) after enrollment.

## Results

**Figure 1:**
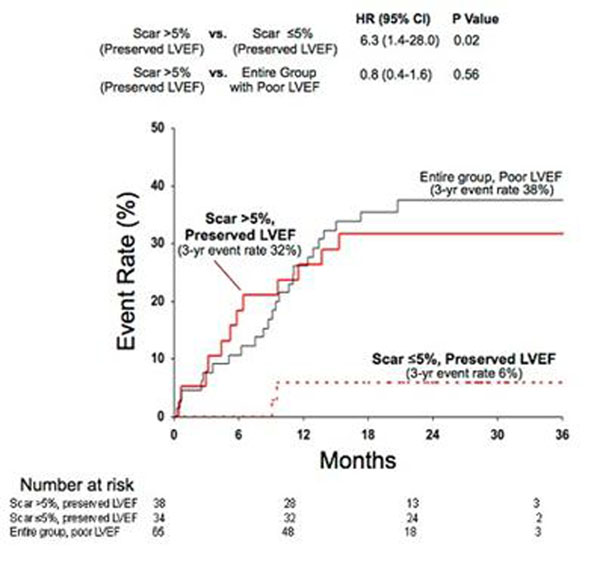


**Figure 2:**
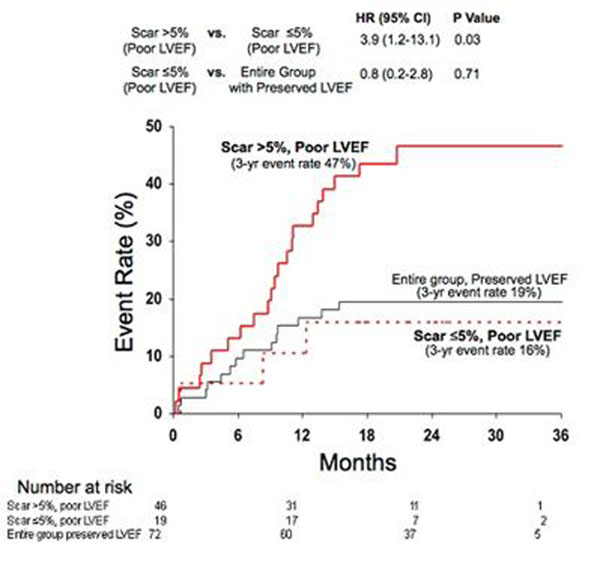


During a median follow-up of 24 months, 39 patients experienced the prespecified primary endpoint of death or appropriate ICD discharge for sustained ventricular tachyarrhythmia. Whereas the rate of adverse events steadily increased with decreasing LVEF, a sharp step-up was observed for scar size >5% of LV mass (HR=5.2 [95% CI, 2.0-13.3]). On multivariable Cox proportional hazards analysis, including LVEF and electrophysiological-study results, scar size (as continuous variable or dichotomized at 5%) was an independent predictor of adverse outcome. Among patients with LVEF >30%, those with significant scarring (>5%) had higher risk than those with minimal-or-no (less than or equal to 5%) scarring (HR=6.3 [1.4-28.0]). Those with LVEF >30% and significant scarring had similar risk to patients with LVEF less than or equal to 30% (p=0.56). (Figure 1) Among patients with LVEF less than or equal to 30%, those with significant scarring again had higher risk than those with minimal-or-no scarring (HR=3.9 [1.2-13.1]). Those with LVEF less than or equal to 30% and minimal scarring had similar risk to patients with LVEF >30% (p=0.71). (Figure 2)

## Conclusions

Myocardial scarring detected by CMR is an independent predictor of adverse outcome in patients being considered for ICD placement. In patients with preserved LVEF, significant scarring (>5% LV) identifies a high-risk cohort similar in risk to those with LVEF less than or equal to 30%. Conversely, in patients with LVEF less than or equal to 30%, minimal-or-no scarring identifies a low-risk cohort similar to those with preserved LVEF.

